# Sublethal exposure to copper supresses the ability to acclimate to hypoxia in a model fish species

**DOI:** 10.1016/j.aquatox.2019.105325

**Published:** 2019-12

**Authors:** Jennifer A. Fitzgerald, Mauricio G. Urbina, Nicholas J. Rogers, Nic R. Bury, Ioanna Katsiadaki, Rod W. Wilson, Eduarda M. Santos

**Affiliations:** a Biosciences, College of Life & Environmental Sciences, Geoffrey Pope Building, University of Exeter, Exeter, EX4 4QD, UK; b Centre for Environment, Fisheries and Aquaculture Science, Barrack Road, The Nothe, Weymouth, Dorset, DT4 8UB, UK; c Departamento de Zoología, Facultad de Ciencias Naturales y Oceanográficas, Universidad de Concepción, Concepción, 4070386, Chile; d University of Suffolk, School of Science, Technology and Engineering, James Hehir Building, University Avenue, Ipswich, IP3 0FS, UK; e Centre for Sustainable Aquaculture Futures, University of Exeter, Stocker Road, Exeter, EX4 4QD, UK; f Instituto Milenio de Oceanografía (IMO), Universidad de Concepción, PO Box 1313, Concepción, Chile

**Keywords:** Teleost, Dissolved oxygen, Metal toxicity, Freshwater, Combined stressors, Critical oxygen level

## Abstract

Hypoxia is one of the major threats to biodiversity in aquatic systems. The association of hypoxia with nutrient-rich effluent input into aquatic systems results in scenarios where hypoxic waters could be contaminated with a wide range of chemicals, including metals. Despite this, little is known about the ability of fish to respond to hypoxia when exposures occur in the presence of environmental toxicants. We address this knowledge gap by investigating the effects of exposures to different levels of oxygen in the presence or absence of copper using the three-spined sticklebacks (*Gasterosteus aculeatus*) model. Fish were exposed to different air saturations (AS; 100%, 75% and 50%) in combination with copper (20 μg/L) over a 4 day period. The critical oxygen level (P_crit_), an indicator of acute hypoxia tolerance, was 54.64 ± 2.51% AS under control conditions, and 36.21 ± 2.14% when fish were chronically exposed to hypoxia (50% AS) for 4 days, revealing the ability of fish to acclimate to low oxygen conditions. Importantly, the additional exposure to copper (20 μg/L) prevented this improvement in P_crit_, impairing hypoxia acclimation. In addition, an increase in ventilation rate was observed for combined copper and hypoxia exposure, compared to the single stressors or the controls. Interestingly, in the groups exposed to copper, a large increase in variation in the measured P_crit_ was observed between individuals, both under normoxic and hypoxic conditions. This variation, if observed in wild populations, may lead to selection for a tolerant phenotype and alterations in the gene pool of the populations, with consequences for their sustainability. Our findings provide strong evidence that copper reduces the capacity of fish to respond to hypoxia by preventing acclimation and will inform predictions of the consequences of global increases of hypoxia in water systems affected by other pollutants worldwide.

## Highlights

• Exposure to hypoxia resulted in acclimation by *G. aculeateus,* demonstrated by a reduction of their critical oxygen level.
• In the presence of copper, the capacity to reduce the critical oxygen tension following hypoxia was prevented.
• Combined exposure of copper and hypoxia resulted in an increase expression of *mt1* and *hif1α* transcription.

## 1 Introduction

Aquatic systems worldwide are facing critical changes in environmental conditions that have the potential to cause shifts in species distributions and extensive local extinctions in poorly connected habitats. Decreases in water pH due to increased atmospheric CO_2_, global increases in temperature and increases in the incidence, severity and prevalence of hypoxia are considered to be the most serious threats to aquatic ecosystems worldwide, including both marine and freshwater systems (Diaz and Breitburg, 2009). It is of critical importance to consider that these stressors rarely occur in isolation, and often occur in areas affected by chemical pollution. Despite this, very little research has been directed to understanding the interactions between these stressors and chemical pollutants, which severely compromise the ability to predict their effects on global aquatic ecosystems. In order to achieve this, knowledge of how stressors interact is required, which can then be used to build predictive models for the impacts of multiple stressors on aquatic life. We aimed to address this challenge by studying the impacts of exposure to reduced oxygen levels in the presence of copper, an aquatic pollutant widespread in freshwaters and considered to be a significant pollutant in many areas, including the UK (Donnachie et al., 2014; Johnson et al., 2017).

Oxygen levels fluctuate in natural environments, and hypoxia occurs when the oxygen concentrations are sufficiently low to become a stressor to organisms living in those systems (Diaz and Breitburg, 2009; Malekpouri et al., 2016). Areas of low oxygen occur naturally in aquatic ecosystems, but in the last decades hypoxic areas have increased rapidly, likely as a result of anthropogenic activity, resulting in extensive hypoxic zones worldwide (Gewin, 2010). Fish cope with hypoxia through a wide range of behavioural, physiological, biochemical and anatomical responses, and tolerance to hypoxia has been shown to vary between species, life stages and habitats (Pollock et al., 2007). Fish attempt to maintain blood oxygen supply by increasing gill ventilation, resorting to air breathing or gill remodelling (Wu, 2002), and acclimate via metabolic suppression, or by reducing activity, feeding, reproduction and growth (Richards, 2009; Wang et al., 2009). At the molecular level, the hypoxia inducible factor (HIF) can sense reduced intracellular oxygen and activate cellular response pathways by regulating the transcription of a number of gene cascades (Zhu et al., 2013). For example, erythropoietin (EPO) transcription has been shown to increase following HIF activation under low oxygen conditions, causing increased red blood cell production and promoting oxygen transport to target tissues (Semenza, 2004).

Low oxygen levels occur regularly in areas prone to discharge of waste waters from industrial and agricultural activities, which contaminate aquatic environments with excess nutrients, resulting in eutrophication and in turn hypoxia. These effluents can contain a wide range of contaminants including toxic metals, such as copper. Copper is a particularly interesting metal because it occurs naturally in the environment and is essential for life, acting as a cofactor for a wide range of enzymatic reactions including those involved in oxygen transport, cellular metabolism and energy production (Kim et al., 2008). However, if the concentrations in the environment exceed certain thresholds, copper can become highly toxic to living organisms. Copper contamination of the aquatic environment is of particular concern because organisms are continuously exposed via the skin and respiratory surfaces, as well as via the diet, including ingestion of contaminated water or sediment (Wood et al., 2012). As a consequence of contemporary and historical mining activities, industrial processes and urban and agricultural runoff, copper concentrations currently reported in the aquatic environment often reach those known to be toxic to fish (Batty et al., 2010). Copper can adversely affect multiple processes including branchial ion transport, haematopoiesis and glycolytic enzymatic activity, and cause immune suppression and oxidative stress (Richards, 2009). In addition, fish exposed to copper have been shown to lose the ability to sense environmental oxygen levels (Geest et al., 2002). This raises a significant concern as aquatic hypoxia and copper pollution often co-occur in the environment (Malekpouri et al., 2016).

Our own studies investigating the combined effects of copper and hypoxia demonstrated that copper toxicity changed dramatically under hypoxic conditions during zebrafish (*Danio rerio*) development (copper toxicity was reduced during early development but increased in hatched larvae) (Fitzgerald et al., 2016), with similar results observed for three-spined stickleback (*Gasterosteus aculeatus*) (Fitzgerald et al., 2017). Combined exposures to copper and hypoxia also increased oxidative stress and caused alterations in blood parameters in the killifish (*Fundulus heteroclitus*) (Ransberry et al., 2016), pacu (*Piaractus mesopotamicus*) (Sampaio et al., 2008) and carp (*Cyprinus carpio*) (Mustafa et al., 2012). For mangrove rivulus (*Kryptolebias marmoratus*), exposure to copper induced hypoxia-like changes to gill morphology and increased the sensitivity of the hypoxia emersion response (Blewett et al., 2017). In addition, for the common carp, a reduction in standard metabolic rate and critical oxygen level (P_crit_) was seen after combined exposure to copper and hypoxia (Malekpouri et al., 2016). Of note, most studies to date were limited to species considered to be relatively tolerant to hypoxia, highlighting the importance of investigating how these combined stressors affect species that have a lower ability to tolerate low oxygen conditions.

In this study we used the three-spined stickleback model to investigate if their response to reduced oxygen in the water was affected by the presence of a sub lethal concentration of copper. The three-spined stickleback is a well-established model in ecotoxicology and we selected it as a model for this study due to its relative intolerance to hypoxia, representing an understudied group of species. We hypothesised that the presence of copper would suppress the natural responses to hypoxia in adult fish, similarly to that previously observed for hatched larvae (Fitzgerald et al., 2017). In order to test this hypothesis, we first determined the sensitivity of the stickleback population to hypoxia by determining their P_crit_, to identify appropriate oxygen concentrations to be used during the copper exposures (above and similar to the Pcrit). We then assessed the effect of exposure to reduced concentrations of oxygen in the water in the presence or absence of copper (20 μg/L; a sub lethal concentration shown to cause measurable responses in this species, and present within polluted environments (Santos et al., 2009)) on a range of physiological and molecular responses following 4 days of exposure. These included changes in Pcrit, as a measure of acclimation to low oxygen, behaviour observations, rates of ventilation, blood parameters (haemoglobin concentrations and haematocrit levels) and the transcription of biomarkers for hypoxia response (*hif1α*), oxidative stress (*cat*) and metal detoxification (*mt*) pathways.

## 2 Material and methods

### 2.1 Fish source, culture and husbandry

A population of freshwater three-spined stickleback (originating from the River Erme, Devon, United Kingdom) was maintained in the Aquatic Resource Centre at the University of Exeter in mixed sex stock tanks (112 L), supplied with aerated synthetic freshwater (Paull et al., 2008). Charcoal-dechlorinated mains tap water was filtered by reverse osmosis (Environmental Water Systems, UK Ltd.) and reconstituted with Analar-grade mineral salts to synthetic freshwater (final concentrations to result in a conductivity of 300 mS; 122 mg/L CaCl_2_2H_2_O, 9.4 mg/L NaHCO_3_, 50 mg/L MgSO_4_7H_2_O, 2.5 mg/L KCl, 50 mg/L), aerated, and maintained at 15 °C in a reservoir before being fed to each tank using a flow-through system. Prior to the experimental period, fish were maintained at a temperature of 15 ± 1 °C and a photoperiod of 18:6 light/dark with 30 min dawn/dusk transitional periods and fed to satiation twice a day with blood worms (*Chironomus sp*.; Tropical Marine Centre, Chorleywood, UK).

### 2.2 Determination of the critical oxygen level (P_crit_)

We conducted a preliminary experiment in which we determined the P_crit_ by semi- closed respirometry for 7 adult male stickleback to provide essential information for the design of subsequent exposure experiments. Individual fish were placed in an experimental glass chamber of 500 ml at 15 °C, each chamber sealed with a rubber bung containing inlets and outlets for two pump controlled systems. In the first system, dissolved oxygen was continually assessed using a flow-through cell (Firesting OXFTC) connected to a FSO2-4 optical oxygen meter (Firesting OXFTC) during set periods of time when oxygen consumption was determined. Water was pumped past the sensor at 50 ml/min, with the sensor measuring the pO_2_ (partial pressure of oxygen) every 2 s. Oxygen electrodes were calibrated daily with fully aerated water (100% oxygen saturation) and a saturated sodium sulfite solution (0% oxygen saturation) (Urbina and Glover, 2013). The second system was a loop to periodically flush the chamber in order to reset the water to the desired pO_2_. After 15 min of continuously measuring the drop in oxygen concentration for determination of metabolic rate (MO_2_), the chambers were flushed with water from a stock tank at the desired pO_2_. MO_2_ measurements were conducted four times at each of the selected levels of air saturation; 100%, 80%, 60%, 40%, 30%, 20%, 15% and 10% (measured level: 100 ± 0.1%, 83 ± 0.9%, 61 ± 0.5%, 43 ± 0.5, 34 ± 0.3%, 24 ± 0.5%, 17 ± 0.4% and 14 ± 0.1%, respectively). To achieve each air saturation level, pO_2_ was controlled by continuous bubbling with a pre-set gas mixture of O_2_, N_2_ and CO_2_ (constant atmospheric level; 0.04%), achieved by controlling the proportional gas flow rates from cylinders of each gas, using precision gas flow controllers (MC Series Mass Flow Controllers, Qubit Systems Inc., Ontario, Canada). The mass flow controllers were connected to a PC running a gas mixing software (C960 Gas Mixing Software, Qubit Systems Inc., Ontario, Canada) in order to set the gas mixtures. To ensure water within each respirometry chamber was well mixed, chambers were placed on a stir plate (Thermo scientific, imareci Poly Komet). A magnetic stir bar was placed at the bottom of each chamber covered by mesh and weighed down by marbles, to prevent the fish from becoming distressed or knocking the stir bar during the experiment. Fish were moved into chambers 24 h before measurements started, to allow for fish to acclimate to the chambers, and return to a stable resting metabolic rate. Throughout the acclimation and experimental periods, fish were not fed.

After the experiment, the P_crit_ was determined by the conversion of each 15 min measurement into the metabolic rate (MO_2_). MO_2_ was calculated using the chamber volume, the slope of oxygen consumption over time (continuous measurement over 15 min), divided by the mass of fish. Regression lines were drawn on the graphs using regression analysis in Excel, and the breakpoint (P_crit_) when there was a sudden change in the response variable (Y: MO_2_) as a function of the independent variable (X: average external pO_2_) was determined. P_crit_ was obtained by determining the intersection of the two linear regression lines (Toms and Lesperance, 2003; Leiva et al., 2015). The measured P_crit_ was 48.88 ± 2.73%.

### 2.3 Combined exposures to copper and varying oxygen concentrations

Adult stickleback males were exposed to 0 or 20 μg/L of copper (added as copper sulphate, Sigma Aldrich), under three different oxygen concentrations: normoxia (100% AS), a moderate decrease in oxygen concentration (75% AS) and hypoxia, corresponding a concentration of oxygen just above the P_crit_ of this species under our experimental conditions (50% AS) from the preliminary P_crit_ determination. Partial pressure of oxygen (pO_2_) was controlled by continuous aerating with a pre-set gas mixture of O_2_, N_2_ and CO_2_ (0.04%) as described above. Chemical exposure was conducted via a flow through system for 4 days (this length of exposure was adopted based on previous studies showing that this exposure length is sufficient to cause measurable changes at the physiological and molecular level (Santos et al., 2009)), with each treatment group comprised of three exposure tanks (40 L) containing 5 fish per tank (n = 15 fish per treatment).

The concentration of oxygen in each tank was measured twice daily to ensure it was kept at the correct level, and daily measurements of conductivity and pH where also conducted. Fish were not fed over the exposure period. On day 1, 2 and 3 of the exposure period, fish were monitored for behavioural effects by recording the position of the fish in the tank. The position of each fish in every tank was scored over a period of an hour, with 6 scores taken per tank. Ventilation rates were also measured on day 1, 2 and 3 of the exposure period by live monitoring for every fish in each tank, by counting the number of times each fish opens its mouth/moves its operculum within a 1 min period.

All fish were sacrificed on day 4 of the exposure period by a lethal dose of benzocaine followed by destruction of the brain, in accordance with the UK Home Office regulations. Wet mass (M) in grams and fork length (FL) in cm were recorded and the condition factor (k = M x 100 / FL^3^) was calculated for each individual fish. Gill and liver samples were taken from each fish, snap frozen in liquid nitrogen and stored at −80 °C for analysis of gene transcription, and placed in acid washed tubes, weighed to determine wet mass of the tissue, then stored at −20 °C for metal analysis.

### 2.4 Metal analysis in water and tissue samples

Water samples were collected from each tank on days 1 and 3 of the exposure period and stored at -20**°**C until chemical analysis. Prior to analysis of total copper concentrations, samples where acidified by adding nitric acid (70%, purified by redistillation, ≥99.999% trace metals basis, Sigma Aldrich) to a final concentration of 0.01% to each sample. Tissue samples were initially freeze dried to determine the dry mass of the samples before acid digestion. Tissue digestion was conducted by adding 500 μl of nitric acid to each tube and incubated at room temperature for 48 h, until all samples had completely digested. 0.1% hydrogen peroxide (Fisher; Hydrogen Peroxide, 100 vol >30%w/v) was added for breakdown and removal of the fatty material. All samples were covered with Parafilm to prevent evaporation. The resulting digested solution was then diluted 1:10 with ultrapure water. The copper content in each water and tissue sample was measured by ICP-MS using a Perkin Elmer NexION 350D instrument running the Syngistix software, v1.0.

### 2.5 Transcript profiling

Real-time quantitative PCR (RT-QPCR) was used to quantify the transcript profiles in the liver and gills of exposed fish (n = 12 per treatment group) for a number of target genes including: metallothionein 1 (*mt1*) a metal binding protein and important biomarker for metal exposure; catalase (*cat*) a gene involved in the response to oxidative stress, and hypoxia inducible factor 1α (*hif1α*) a gene involved in the response to hypoxia. Three control genes were included in this study: ribosomal protein l8 (*rpl8*), ubiquitin (*ubi*) and beta-tubulin (*tubb4*). Ct values for each sample were plotted, and regression analysis was carried out to identify the most appropriate control gene, classified as the gene that remained the most stable across all exposures, which resulted in the selection of *rpl8* for the analysis. RT-QPCR assays for each target gene were optimised as previously described (Uren Webster et al., 2014) (detailed information of assays is presented in Table S1).

Total RNA was extracted from tissues samples using the Tri reagent method (Sigma-Aldrich, UK) according to manufacturer’s instructions. The concentration and purity of the resulting RNA was determined using a NanoDrop-1000 Spectrophotometer (NanoDrop technologies, Wilmington, USA). One μg of total RNA was subjected to DNase treatment (RQ1 DNase, Promega, Southampton, UK) to remove potential DNA contamination, prior to being converted to cDNA using M-MLV reverse transcriptase (Promega, UK) and primed with random hexamers (MWG-Biotech), according to the manufacturer’s instructions (MJ Research PTC200 Thermal Cycler). cDNA was diluted (1:2), then RT-QPCR was performed using an iCycler iQ Real-time Detection System (BioRad Laboratories, Hercules, USA) and SYBR green chemistry (BioRad Laboratories, Hercules, USA). Each sample was amplified in duplicate in 96-well optical plates (BioRad Laboratories, Hercules, USA) in 15 μl reaction volumes. Efficiency-corrected relative transcription levels were determined using the 2-^ΔΔ^CT method (Livak and Schmittgen, 2001; Filby and Tyler, 2005). Samples where amplification was not detected were excluded from analysis, resulting in n = 8–12 individuals per treatment group.

### 2.6 P_crit_ determination and measurement of blood parameters in fish after exposure to copper at varying oxygen concentrations

The P_crit_ was measured after exposure to copper in combination with different oxygen levels, as described above. Eight fish where exposed to either 100 or 50% air saturation in combination with 0 or 20 μg/L of copper, as described above. On day 3 of exposure, fish where moved into individual glass respirometry chambers containing the same water conditions as the exposure tanks, for acclimation. Oxygen consumption rates were measured from the beginning of acclimation, and throughout day 4, over 20 min periods, followed by decreasing oxygen levels after 80 min intervals (i.e. 4 separate measurements of oxygen consumption for each fish at each oxygen level). The oxygen consumption rates obtained during the period immediately prior to the initiation of the P_crit_ measurements were used to calculate the average metabolic rate under our experimental conditions.

In order to validate the P_crit_ measurements and to ensure no human bias on the analysis of the data, the P_crit_ was calculated using both the broken stick method (as described in the Supporting information) and the Yeager free software (Yeager and Ultsch, 1989), and both methods resulted in similar results (for the control group, using the broken stick method: P_crit_ 54.64 ± 2.51% AS; and using the Yeager software: P_crit_ was 50.24 ± 2.95% AS; P = 0.15). Further, we performed a closed respirometry test where oxygen levels inside the chambers were reduced by the oxygen consumption of the fish, to ensure our system was measuring the P_crit_ appropriately, and stress from the set up was not affecting the results. Dissolved oxygen was continuously assessed using a needle-type fibre optic sensor (Firesting OXR 230) connected to a FSO2-4 optical oxygen meter, and the P_crit_ was calculated using both methods again (broken stick method: P_crit_ 51.66 ± 2.39% AS; Yeager software: P_crit_ was 54.27 ± 3.80% AS; P = 0.74), validating the measurements obtained with the semi-closed system. Based on the results from the validation experiments, throughout this study we adopted the first method (broken stick method) for all P_crit_ measurements.

After the exposure, tissues were sampled as described above, and blood was sampled for haematocrit (relative volume of red blood cells (RBC) compared to whole blood) and blood haemoglobin content measurements (Urbina and Glover, 2012). Haemoglobin was measured by adding 2 μl of blood extracted from each fish to 500 μl of Drabkin’s reagent (Sigma), and the absorbance of samples were read at 540 nm on a spectrophotometer (Teacon i control). Haematocrit and haemoglobin from additional control fish (that had not been placed under exposure conditions, for either copper, reduced air saturation, or P_crit_ measurements; n = 8) were also measured to verify that the short term hypoxia that the fish experienced during the P_crit_ measurements had no effect on the results.

### 2.7 Statistics

Gene transcription data was first scrutinised by the Chauvenet’s criterion to detect outliers for each gene and these were subsequently removed before analyses (Chauvenet, 1863). Data that did not meet the normality (Sharpo-Wilko test) and equal variance (Bartlett test) assumptions was log transformed before analyses. All data were analysed using an analysis of variance model using the software package “R” (Team, 2012). A separate model was used for each dataset, to test for effects of exposure to copper, air saturation (categorical variables) and the interaction between these variables. Minimum adequate models were derived by model simplification using F tests based on analysis of deviance (Crawley, 2012). Tests reported refer to the significance of removing terms from the models. When a significant effect of interaction was identified, pairwise comparisons to determine which groups differed were conducted using Tukey’s HSD post hoc test. All data were considered statistically significant when p<0.05.

## 3 Results

### 3.1 Water chemistry and morphometric parameters

For both the main exposure and the P_crit_ determination, the mean measured concentration of copper in the tank water was 22.14 ± 0.42 μg/L for copper treatments and 0.47 ± 0.01 μg/L for treatments without copper, and a detailed breakdown is presented in the Supporting information Table S2. The mean measured oxygen levels for the three treatments were 97.3 ± 0.3, 76.8 ± 0.3 and 55.2 ± 0.9% of air saturation. For both copper and AS, given that the measured concentrations are close to the nominal concentrations, we refer to the nominal concentrations throughout the manuscript. The average conductivity (229.7 ± 0.5 μS) and pH (7.48 ± 0.02) measured in the tanks throughout the experiment remained stable and were not affected by the treatments.

Throughout the exposure, fish remained in good condition and no mortalities were recorded. The mean body mass, length and condition factor of the fish were 1.58 ± 0.03 g, 5.55 ± 0.03 cm and 0.92 ± 0.01, respectively, and there were no significant differences between treatment groups.

### 3.2 Effects of exposure to varying concentrations of oxygen in the presence or absence of copper on fish behaviour and ventilation rates

There was no significant effect of treatment on fish behaviour (P = 0.900; Fig. S1), although there was a significant effect of position with the majority of fish occupying primarily the bottom third of the tank (P < 0.001; Fig. S1), a typical behaviour for this species.

**Fig. 1:**
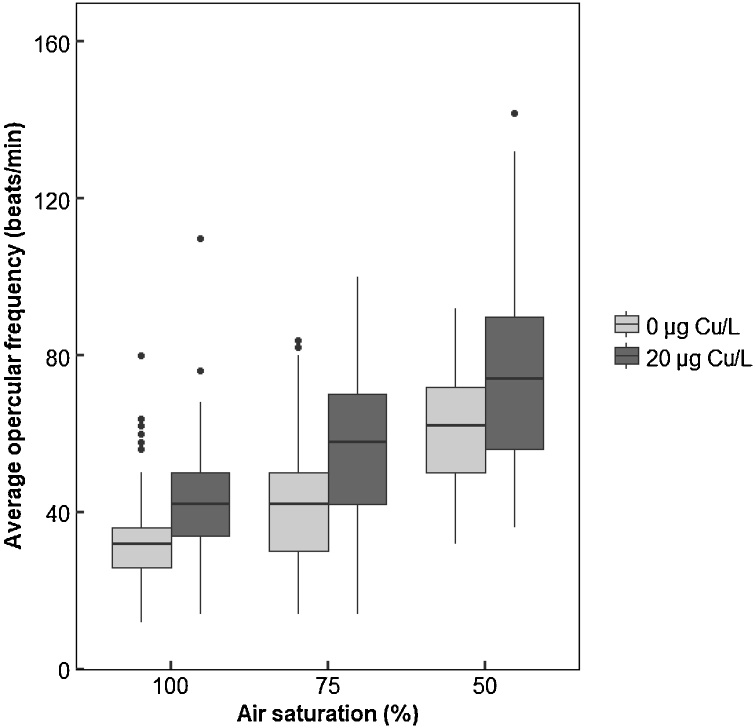
Measured ventilation rates (number of beats of the operculum over one minute) in male sticklebacks exposed to different levels of air saturation (100%; 75% and 50% AS) in the presence or absence of copper (0 or 20 μg Cu/L). Each treatment consisted of 3 tanks containing 5 fish per tank, and measurements were taken over the first 3 days of exposure. Statistical comparisons were conducted using accepted minimum adequate models (analysis of variance model, R; P < 0.05) with model details reported in Table 1.

With decreasing oxygen levels both in the presence and absence of copper, there was a significant increase in ventilation rate (P < 0.001; Fig. 1; Table 1). For all oxygen levels, fish exposed to 20 μg/L of copper had a significantly increased ventilation rate compared to fish exposed to the same level of oxygen in the absence of copper (P < 0.001; Fig. 1).

**Table 1:** Analysis of variance models for the relationships between copper concentration, air saturation and the copper concentration/air saturation interaction. Minimum adequate models (F value) for ventilation rate, metal content, *mt1* transcription, *cat* transcription, *hif1-α* transcription, P_crit_ and blood parameters are shown (Significance codes: *** P < 0.001, ** P < 0.01, * P < 0.05).

		df	Minimum Adequate Model		
			Copper Conc.	Air Saturation	Copper Conc. / Air Sat. Interaction
**Ventilation Rate**		311	37.44***	85.46***	NS
**Metal Content**	**Gill**	42	212.63***	NS	NS
	**Liver**	NS	NS	NS	NS
***mt1* transcription**	**Gill**	72	63.42***	15.06***	NS
	**Liver**	79	26.94***	NS	NS
***cat* transcription**	**Gill**	NS	NS	NS	NS
	**Liver**	NS	NS	NS	NS
***hif-1α* transcription**	**Gill**	76	12.61***	4.07*	NS
	**Liver**	63	12.48***	8.07***	NS
**P_crit_**		23	0.84	6.14*	10.01**
**Blood Parameters**	**Haematocrit**	30	NS	10.01**	NS
	**Haemoglobin**	NS	NS	NS	NS

### 3.3 Copper content in fish tissues

**Fig. 2:**
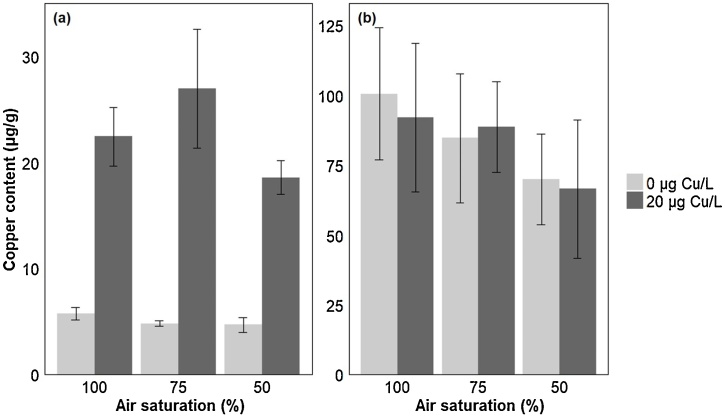
Measured copper concentrations (mean μg Cu/g ± standard error mean) in (a) Gill and (b) Liver of male sticklebacks exposed to different levels of air saturation (100%; 75% and 50% AS) in the presence or absence of copper (0 or 20 μg Cu/L) (n = 7/8 fish for each treatment group). Data are presented as average +/- standard error of the mean. Statistical comparisons were conducted using accepted minimum adequate models (analysis of variance model, R; P < 0.05) with model details reported in Table 1.

In the gill, there was an approximately 5-fold increase in the copper concentration in fish exposed to copper, compared to non-exposed controls (P < 0.001; Fig. 2a; Table 1), and this was not affected by the level of oxygen in the water (P = 0.120). However, there was no effect of oxygen level or copper exposure on the copper content measured in the liver of exposed fish (P = 0.938; Fig. 2b; Table 1).

### 3.4 Transcript profiling for molecular biomarkers

**Fig. 3:**
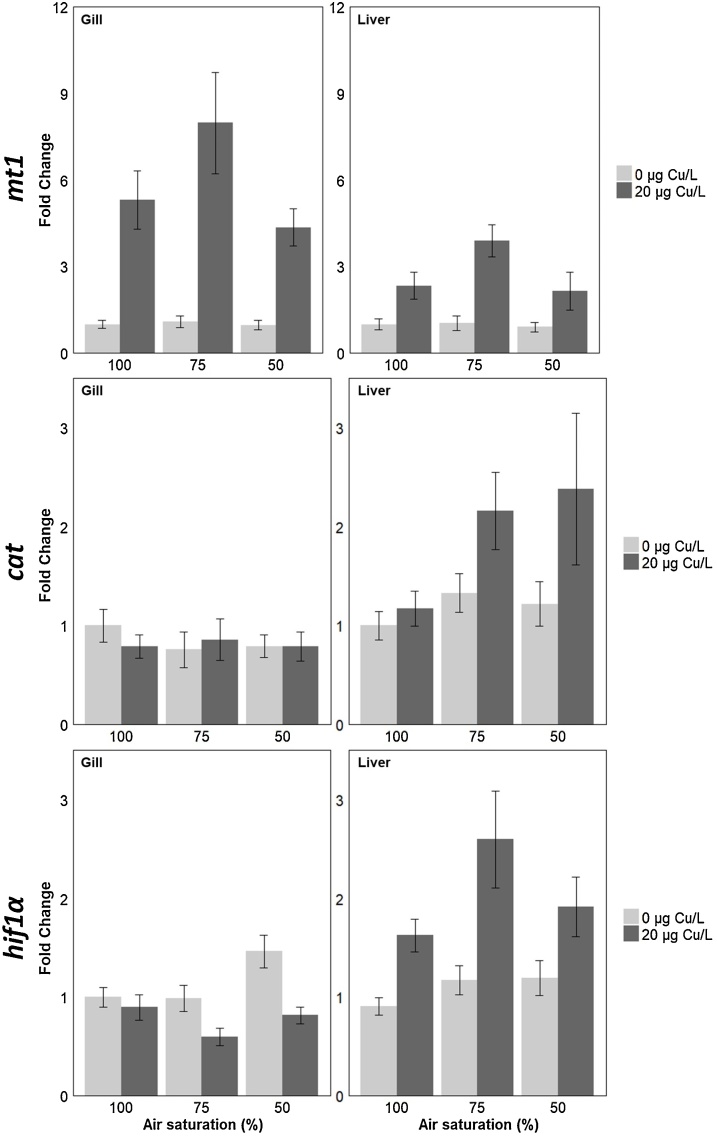
Transcript profiles for target genes (metalothionein 1 (*mt1*), catalase (*cat*), and hypoxia inducible factor 1α (*hif1α*)) in the gill or liver of male sticklebacks exposed to different levels of air saturation (100%; 75% and 50% AS) in the presence or absence of copper (0 or 20 μg Cu/L). Data are presented as average fold change ± standard error mean, with the transcription of each gene normalised against the transcription of the control gene *rpl8*. Individual data points classified as outliers, identified by Chauvenet’s criterion, and points for which the transcription was below the detection limit of the assay were excluded from the analysis, resulting in a replication of n = 8–12 fish per treatment group. Statistical comparisons were conducted using accepted minimum adequate models (analysis of variance model, R; P < 0.05) with model details reported in Table 1.

The transcription of the metal binding protein, *mt1*, was significantly up-regulated in the gills of fish exposed to copper for all oxygen levels tested (P < 0.001; Fig. 3; Table 1). In addition, there was a significant effect of oxygen on the transcription of *mt1* (P < 0.001). In the liver, a similar trend was observed but there was no significant effect of oxygen saturation in *mt1* transcription (P = 0.087) (Fig. 3). In contrast, for *cat* transcription, no changes associated with any of the exposure conditions were observed in either of the tissues analysed (Gill: P = 0.569, and Liver: P = 0.051; Fig. 3; Table 1).

For *hif1α* transcription, significant effects were observed in the liver and gill of exposed fish (Fig. 3). In the gill, there was a significant effect of the oxygen saturation (P = 0.02; Fig. 3; Table 1) and copper exposure (P < 0.001; Fig. 3; Table 1). In the liver, *hif1α* was significantly upregulated in fish exposed to copper compared to the controls (P < 0.001; Fig. 3; Table 1) and there was also a significant effect of oxygen saturation on *hif1α* transcription (P < 0.001; Table 1).

### 3.5 Effects of exposure to varying concentrations of oxygen in the presence or absence of copper on P_crit_

**Fig. 4:**
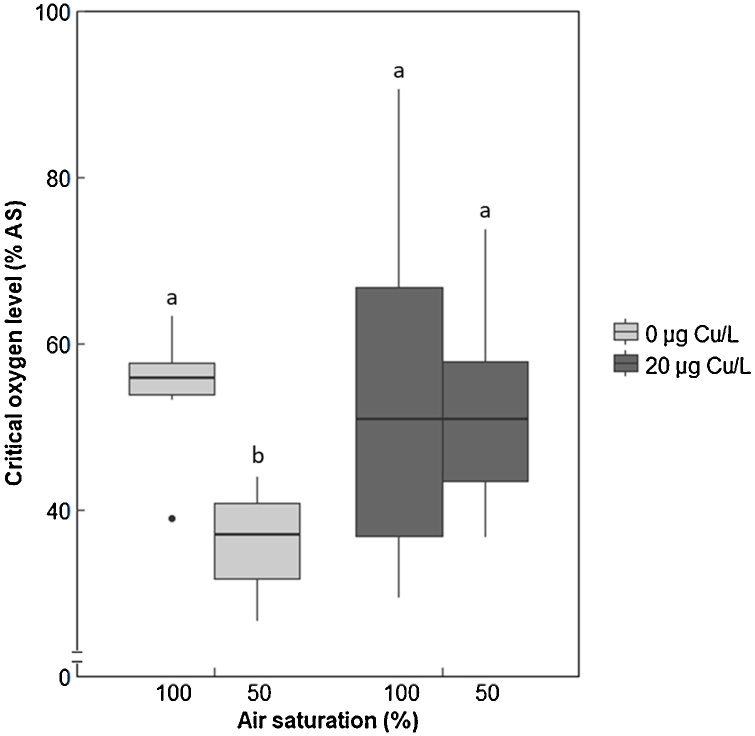
Critical oxygen levels (P_crit_) measured for sticklebacks following exposure to different levels of air saturation (100% and 50% AS) in the presence or absence of copper (0 or 20 μg Cu/L) (n = 8 fish per treatment). Statistical comparisons were conducted using accepted minimum adequate models (analysis of variance model, R; P < 0.05) with model details reported in Table 1.

Exposure to 50% air saturation in the absence of copper resulted in a decreased P_crit_ compared to fish maintained under normoxia (P < 0.003; Fig. 4). In contrast, in the presence of copper at this same oxygen level, no changes in P_crit_ were observed (P = 0.941; Fig. 4). Similarly, the average metabolic rate was decreased in fish exposed to hypoxia compared to fish kept in control conditions, in the absence of copper (P < 0.001; Fig. S2). In the presence of copper, this parameter did not differ as a result of changes in air saturation (P = 0.143; Fig. S2).

**Fig. 5:**
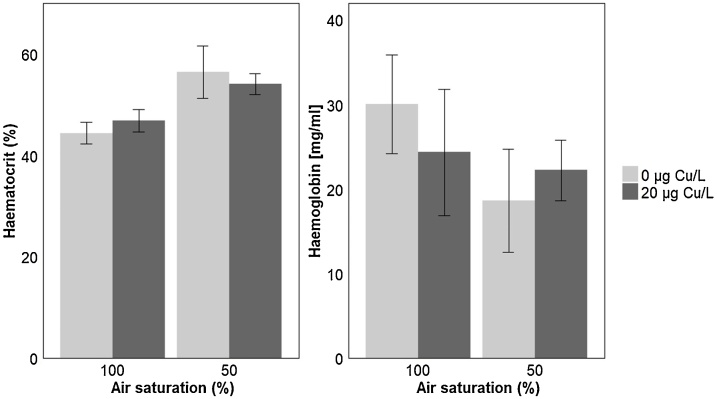
Measured (a) haematocrit and (b) haemoglobin concentration from stickleback exposed to different levels of air saturation (100% or 50% AS) in the presence or absence of copper (0 or 20 μg Cu/L) (n = 8 fish for each treatment group). Data are presented as average ± standard error mean. Statistical comparisons were conducted using accepted minimum adequate models (analysis of variance model, R; P < 0.05) with model details reported in Table S3.

For haematocrit measurements, we first compared control fish that had undergone P_crit_ measurements to those that had not been subjected to this procedure to investigate if the procedure of measuring the P_crit_, which involves progressively reducing oxygen levels in the water, had caused a significant difference in this parameter, and no significant difference was found (P = 0.061). There was a significant increase in the haematocrit in fish exposed to hypoxia for 4 days compared to fish exposed to normoxia (P = 0.004; Fig. 5a; Table 1), however there was no effect of copper on the haematocrit levels (P = 0.773). In addition, there was no significant effect of oxygen or copper treatment on blood haemoglobin content (P = 0.240; Fig. 5b; Table 1).

## 4 Discussion

The objective of this study was to determine if the responses to low oxygen availability in the water are affected by the presence of copper in three-spined stickleback exposed to these combined stressors. Fish were able to lower their P_crit_ in response to exposure to hypoxic conditions, as part of a normal acclimation response to decreased oxygen concentrations in the water, but this ability was no longer evident in fish simultaneously exposed to copper and hypoxia. P_crit_ is a whole organism performance indicator that represents the integrated effects on multiple molecular, biochemical and physiological systems, and it serves as a useful measure of phenotype that is linked to fitness in the wild (Rogers et al., 2016). The lack of ability to reduce the P_crit_ when fish are exposed to hypoxia in the presence of copper suggest that adult sticklebacks can lose their ability to acclimate to hypoxia and are at an increased risk of adverse effects resulting from the combined exposures to these stressors compared to the risk predicted based on data for each stressor alone.

### 4.1 Responses of stickleback to copper exposure in the presence or absence of reduced oxygen concentration

Copper content was strongly increased by 3–5 fold in the gills of fish exposed to copper compared to fish maintained under control conditions for all levels of oxygen tested. These data were mirrored by the induction of *mt1* transcription, a key biomarker for metal toxicity. *mt1* is a metal binding protein involved in the storage and detoxification of metal ions through binding and removal of their redox potential. Metallothioneins account for a major proportion of the cellular storage of copper in fish (Egli et al., 2006; Wood et al., 2012), with increases in *mt* transcription shown to occur in response to metals, in both short and long term exposures, and in chronically exposed wild fish (Hansen et al., 2006; Hogstrand and Haux, 1991; Uren Webster et al., 2013). Although this transcript is predominately associated with the liver, its presence and responsiveness to metals has also been documented in the gills for a range of species (van Heerdan et al., 2004; Uren Webster et al., 2013; our own unpublished data).

The gills are a major site of copper uptake from the ambient water in freshwater fish (Grosel and Wood, 2002). Copper is exported from the gill cells to the blood and transported to the liver, a key organ for copper homeostasis. In the liver, copper is incorporated into ceruloplasmin transcuprein and also bound to albumin to be exported and used elsewhere in the body (Craig et al., 2007). Alternatively, in the liver copper can be sequestered by metal chaperone proteins, for detoxification and storage, and ultimately to be excreted via the bile (Craig et al., 2007). In our study there was no observable increase in copper content in the liver. This is surprising and the reasons for this observation are unclear. We measured an induction in *mt1* transcription in the liver of fish exposed to copper compared to the controls, suggesting an initiation of a molecular response to elevated copper in liver cells. Therefore, we hypothesise that in our experimental conditions, the expected accumulation of copper in the liver may have been initiated, but not to a detectable level, using the measurements employed in this study.

No significant changes in *cat* transcription were observed in the gills or the liver of fish exposed to copper. This lack of changes observed may be as a result of intracellular copper being bound to metallothionein or other metal binding proteins and not in its biologically active form, reducing its toxicological effects within the cell. In a previous exposure to copper in three-spined stickleback, glutathione levels and antioxidant enzymes in the liver returned to a basal level despite copper accumulation, as a result of metallothionein binding capabilities (Sanchez et al., 2005), supporting the present observations for *cat*. Of note, our measurements were limited to the transcriptional level, and therefore they do not exclude changes in the activity of this enzyme, but they indicate that *cat* transcriptional regulation was not affected by the exposure.

The hypoxia inducible factor (HIF) pathway is essential for the response to low oxygen within the cell but has also been shown to be induced following some chemical exposures (Richards, 2009). For both the gill and liver, reduced air saturation caused a significant increase in *hif1α* transcription but, in contrast, copper caused a marked increase in *hif1α* transcription in the liver but a suppression of transcription in the gill. It has previously been demonstrated that exposure to copper in rainbow trout (*Oncorhynchus mykiss*) induced HIF1α protein in the gills (Heerden et al., 2004). Work by Martin et al., 2005 demonstrated that copper can stabilise the nuclear HIF1α under normoxic conditions, through PHD/HIF/HRE-dependent gene regulation, serving as a sensory system not only for oxygen but also for copper (Martin et al., 2005). Using *in vitro* hydroxylation assays, copper inhibited prolyl-4-hydroxylation independently of iron and oxygen concentration. The authors suggested that this could mean that increased concentration of free copper regulates its binding protein, ceruloplasmin, in the liver via a HIF-dependent mechanism (Martin et al., 2005). This was further supported by observations *in vivo* showing an increase in HIF target genes as a result of copper exposure, including plasma ceruloplasmin, after copper was administered intravenously to copper-deficient pigs (Roeser et al., 1970) and the increase in VEGF at wound sites treated with copper sulphate (Sen et al., 2002). Therefore, it is likely that in our study copper could be directly regulating *hif1α* transcription. It is unclear why copper caused opposite effects on *hif1α* transcription in the gill and the liver, but this could be related to the increased concentration of copper in the gills, and absence of significant copper accumulation in the liver. Further studies are needed to evaluate how copper affects this gene signalling pathway using global approaches to provide an unbiased view of its global effects.

### 4.2 Response to decreased oxygen concentration was suppressed in the presence of copper

An increase in ventilation rates was observed in fish exposed to decreased oxygen concentrations. Hyperventilation is a common response to low oxygen in fish, as it allows the maintenance of the same rate of oxygen uptake and delivery to cells despite declining oxygen availability in the water. This is an important physiological response as increases in ventilation volume driven by changes in breathing frequency or amplitude help maintain arterial oxygen levels which in turn delay the switch from aerobic to anaerobic metabolism (Perry et al., 2009).

Copper significantly increased the ventilation rate of exposed fish for all air saturation levels tested. This has been reported in other species, including in trout where exposure to copper resulted in reduced swimming activity and increased ventilation rates (Beaumont et al., 1995), and in the flying barb (*Esomus danricus*) where exposure to copper resulted in an increase in ventilation frequency (Vutukuru et al., 2005). Increased ventilation rates are considered to be linked to the physiological stress induced by copper toxicity. For example, copper exposure can induce mucous secretion, inflammatory responses and changes in gill cell morphology, which will likely increase barriers for gas diffusion, requiring a compensatory increase in ventilation rate (Arellano et al., 1999; De Boeck et al., 2001; Figueiredo-Fernandes et al., 2007; Shephard, 1994; van Heerden et al., 2004). Importantly, the combined exposure to reduced oxygen and copper resulted in a greater increase in ventilation rate compared to that observed following exposure to the individual stressors alone, for both 75% and 50% AS. Increasing the ventilation rate incurs a metabolic cost and there is usually a limit for how much a fish may increase its ventilation rate before reaching a threshold where this adaptation is no longer beneficial (Perry et al., 2009). Therefore, this supports the hypothesis that combined exposures to hypoxia and copper result in greater respiratory stress and greater metabolic costs compared to exposures to each stressor alone.

The P_crit_ is a useful measure of hypoxia tolerance in fish because it reflects the capacity to maintain aerobic metabolism in low pO_2_ environments. It is defined as the point at which an organism transitions from being an oxyregulator to an oxyconformer (Chapman et al., 2002), and this has recently been reviewed for 151 teleost species (Rogers et al., 2016). The P_crit_ measured for fish in or study under normoxia was broadly in line with previous reports for this species (Regan et al., 2017). We used this measure to assess if exposure to copper, either alone or in combination with hypoxia, would affect hypoxia tolerance in adult sticklebacks. When sticklebacks were maintained under hypoxia for 4 days, a decrease in their P_crit_ was observed, reflecting their ability to acclimate to the reduced concentrations of oxygen in the water. Upon exposure to hypoxia, immediate survival is dependent on the fish’s ability to maintain metabolic function through the modification of existing physiological and biochemical pathways. In general, the first response to hypoxia involves attempting to maintain oxygen delivery by increasing water flow over the gills (an increased ventilation rate observed in our study confirms the presence of this mechanism), or by increasing the diffusion capacity through the gill and the conservation of energy expenditure (through the reduction in general metabolism; verified in our study as there was a decrease in the metabolic rate of fish exposed to hypoxia; down regulation of protein synthesis, and/or through the reduction of locomotor activities) (Wu, 2002). If the fish are successful with the immediate responses to the hypoxic conditions, they will then acclimate over time (Richards, 2009). This process involves both physiological processes and changes in transcription, resulting in functional changes of hypoxia specific pathways that will help the fish to survive and function under low oxygen conditions (Richards, 2009).

An important element in the process of acclimating to hypoxia involves supporting oxygen delivery in the face of reduced environmental availability, which involves the modification of haemoglobin-oxygen binding affinity and blood haemoglobin content. In our study, blood haematocrit levels were significantly increased in fish maintained under hypoxic conditions compared to normoxia, independent of whether they were exposed to copper or not. However, there were no significant alterations in the concentration of haemoglobin as a result of hypoxia or copper exposure, despite the increase in haematocrit. This is likely to be as a result of the red blood cells absorbing more water and swelling under hypoxic conditions, and not as a result of an increased number of red blood cells in the circulation. This effect has been documented as a response to both general stress and exposure to hypoxia in fish (Cossins, 1989): increases in cell volume as a result of hypoxia exposure are thought to be a consequence of erythrocyte pH regulation which involves the activation of sodium/proton exchange across the cell membrane (Nikinmaa, 2001). The resulting increase in cellular Na^+^ content, drawing in water by osmosis, simultaneously elevates red blood cell pH and thereby the haemoglobin-oxygen affinity. This allows the blood to bind oxygen more effectively at the gills and therefore contributes to acclimation to low oxygen.

A critical finding in our study was that exposure to copper prevented fish from being able to reduce their P_crit_ in response to exposure to decreased oxygen in the water, as observed in fish exposed to decreased oxygen alone (discussed above). To our knowledge this is the first time that copper exposure has been shown to affect the ability of the fish to respond to low oxygen conditions. Interestingly, this effect occurs together with an increase in ventilation rates observed for fish exposed to copper and reduced oxygen concentration simultaneously, illustrating the increased demand for obtaining oxygen under these conditions. Further, this is supported by the fact that in groups exposed to copper, a decrease in oxygen concentration in the water did not affect the metabolic rate of exposed fish. Work by Malekpouri et al. (2016) reported a different trend when they investigated the effects of the combined exposure to copper and hypoxia on the metabolic capabilities of the crucian carp (Malekpouri et al., 2016). They found that the interaction of the two stressors caused the maximum metabolic rate and aerobic scope to increase after a chronic 7 day exposure but the P_crit_ was reduced. The crucian carp is extremely tolerant to hypoxia, being able to survive even in anoxic conditions for extended periods of time (Figueiredo-Fernandes et al., 2007). To survive such low levels of oxygen, this species has developed adaptive strategies, including the ability to produce ethanol as the end-product of anaerobic metabolism, adjustment of the gill morphology to help supply its oxygen needs, haemoglobin with extremely high oxygen affinity and mechanisms of neural depression (Figueiredo-Fernandes et al., 2007). Therefore, it is perhaps unsurprising that carp are able to reduce their P_crit_ even when exposed to combinations of copper and hypoxia, as they have an extensive array of mechanisms of adaptation to low oxygen levels, especially compared to the stickleback that is considered a relatively hypoxia sensitive species (Leveelahti et al., 2011; O’Connor et al., 2011; and supported by the relatively high P_crit_ found in the present study). This emphasises why it is important to study fish with different tolerances to hypoxia when investigating how fish respond to combinations of hypoxia with other stressors, as hypoxia tolerance could be a strong determinant of their ability to respond to combined exposures, in particular for chemicals that share pathways of effect with hypoxia.

Of note, the variability in the P_crit_ between individual fish appear to be greater in the groups exposed to copper compared to the control groups, independent of whether they were exposed to hypoxia. Similarly, an apparent increase in variability occurred in the measured P_crit_ in the Crucian carp when exposed to copper (Schjolden et al., 2007). This suggests that individuals within the population may have different thresholds of response, and therefore for wild populations exposed to these stressors in combination, it is possible that a selection for a tolerant phenotype may occur, potentially leading to alterations in the gene pool of populations. Such changes in the genetic makeup of populations may render them vulnerable to other stressors by reducing their ability to adapt to changing environmental conditions. Together, our data highlight the complexities of predicting the effects of multiple stressors in complex environments, where individual variation is critical to population survival.

Interestingly, fish appeared to have a greater molecular response to copper at the intermediate level of oxygen (75% AS) compared to the lowest (50% AS), with an apparent greater induction in the transcription of *hif1α* and *mt1*. The lowest level of oxygen used for the 4 day exposures (50% AS) corresponded to a level close to the critical oxygen level (P_crit_) for this population, so the fish were likely to be under significant stress and on the edge of maintaining their oxygen supply in an oxygen depleted environment, which may have resulted in a reduced ability to increase gene transcription. This reduced transcriptional response observed in fish exposed to copper and 50% AS compared to those exposed to copper and 75% AS supports the hypothesis that their response to combined copper and hypoxia exposures is suppressed, with potential impacts to their ability to acclimate to these conditions.

## 5 Conclusions

We have demonstrated that the expected decrease in P_crit_ in a teleost species exposed to low oxygen conditions was abolished in the presence of a toxic metal (copper), removing the acclimation process to decreased oxygen, which is essential to survival in hypoxic waters. In addition, the individual variation in P_crit_ measurements increased in groups of fish exposed to copper, with potential consequences for selection of tolerant phenotypes and loss of genetic variability if such effects were to occur in wild populations. Our data contrasts with previous studies for carp and demonstrate the importance of considering species with a range of hypoxia tolerance when investigating the effects of chemical toxicity on the responses to hypoxia, as they could respond differently to combinations of these stressors. Considering the strong likelihood that hypoxic events co-occur with other stressors, including chemical pollutants, it is fundamental to understand the interactions between the effects of these combinations of stressors. These mechanistic studies will provide the knowledge base necessary to develop modelling approaches capable of predicting the consequences of exposures for fish populations and management decisions aimed to protect the natural environment.

## Funding

We acknowledge support for this work from a PhD studentship supporting J. A. Fitzgerald, funded by the University of Exeter and the Centre for Environment Fisheries and Aquaculture Science (Cefas), as part of their Strategic Alliance. M. G. Urbina was funded by a BBSRC grant (BB/J00913X/1) to R. Wilson and by the CONICYT-FONDECYT Grant11160019.

## Authors’ contributions

J.A.F., M.G.U., R.W.W. and E.M.S. conceived the project and designed the experiments. I.K. advised on the project. J.A.F. carried out the experiments, N.J.R and M.G.U. assisted with the Pcrit experiments. N.R.B carried out ICP-MS analysis. J.A.F and E.M.S. wrote the manuscript, and all authors edited and approved the final manuscript.

## Declaration of Competing Interest

The authors declare that they have no known competing financial interests or personal relationships that could have appeared to influence the work reported in this paper.
