# Supplementary material for: Sublethal exposure to copper supresses the ability to acclimate to hypoxia in a model fish species
Source: Aquat Toxicol. 2019 Dec;217:105325. doi: 10.1016/j.aquatox.2019.105325 (PMC6891231; doi:10.1016/j.aquatox.2019.105325)
Supplement: Supplementary file 1 [file mmc1.docx]

**Supplementary Information:**

**Sublethal exposure to copper supresses the ability to acclimate to hypoxia in a model fish species**

*Jennifer A. Fitzgerald, Mauricio. G. Urbina, Nicholas J. Rogers, Nic R. Bury, Ioanna Katsiadaki, Rod W. Wilson, Eduarda M. Santos*


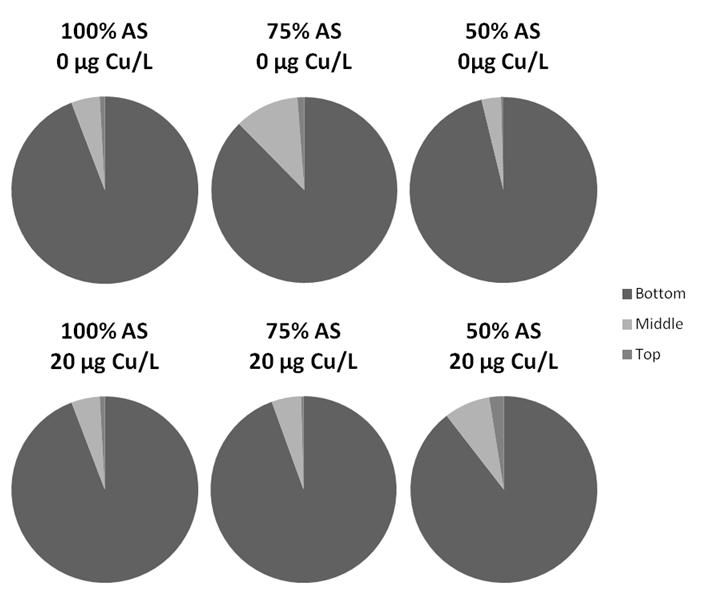


**Figure S1**. Pie charts documenting the position in the tank of fish over the exposure period. Male sticklebacks were exposed to 0 or 20μg Cu/L for 4 days under different levels of air saturation (100%; 75% and 50% AS). Each treatment consisted of 3 tanks containing 5 fish, and the position in the tank of each individual fish for the first 3 days of exposure was recorded. Statistical comparisons were conducted using accepted minimum adequate models (analysis of variance model, R; P<0.05), where no significant effect between the different treatments occurred (P=0.900).


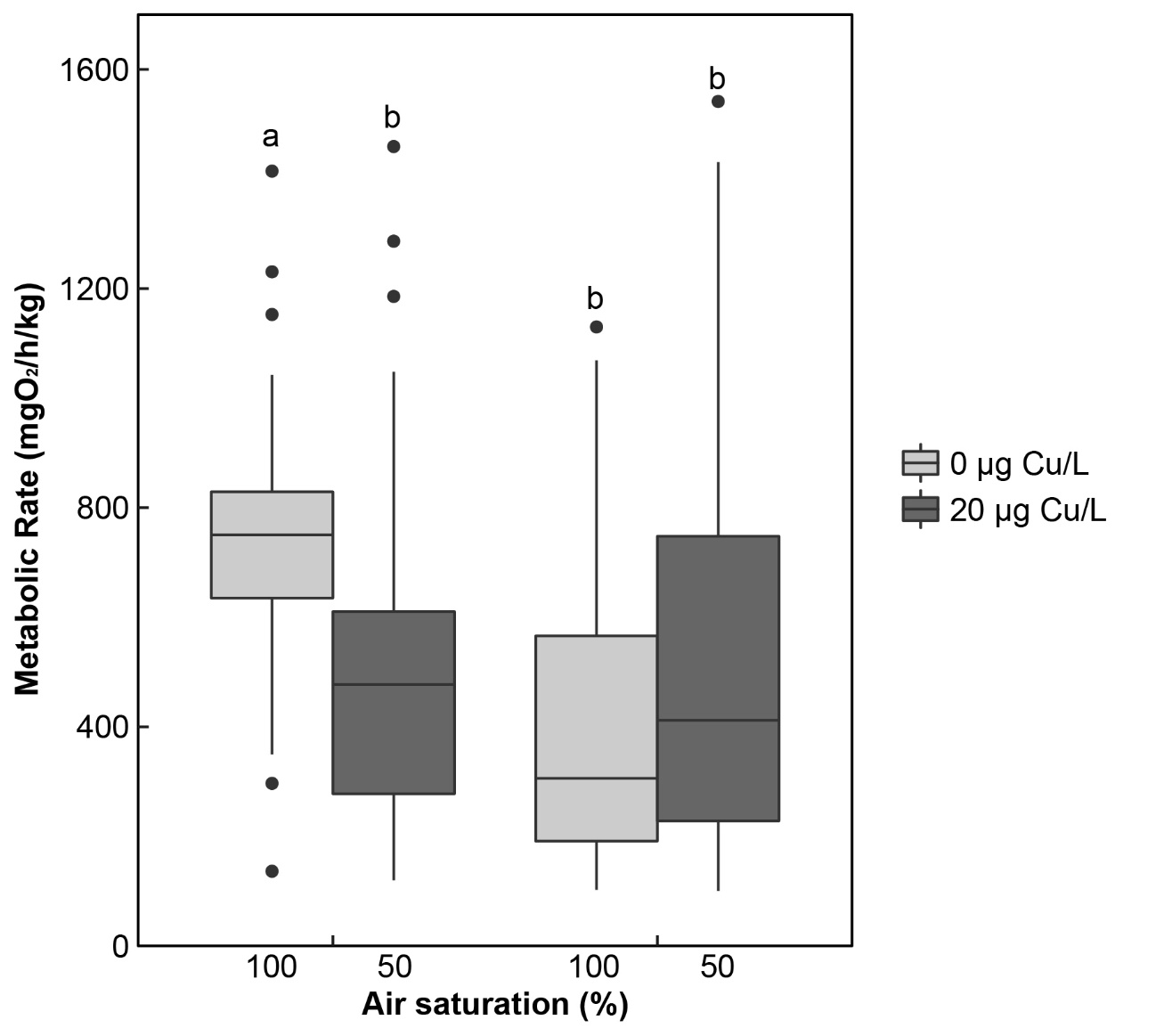


**Figure S1**. Average metabolic rate (mgO_2_/h/kg) measured for individual stickleback immediately prior to the Pcrit measurements following exposure to different levels of air saturation (100% and 50% AS) in the presence or absence of copper (0 or 20 μg Cu/L; n=8 fish per treatment). Statistical comparisons were conducted using accepted minimum adequate models (analysis of variance model, R; P < 0.05), with a significant interaction occurring between air saturation and copper treatment (P < 0.001), therefore letters above the graph detail the between group comparison (Tukey Multiple Comparison).

**Table S1**: Real Time Quantitative PCR assay details. Primer sequences, amplicon product size, annealing temperature and PCR efficiency for each target gene is presented.

| **Gene Name** | **Gene Symbol** | **Forward Primer (5’-3’)** | **Reverse Primer (5’-3’)** | **Product size (bp)** | **Ta (°C)** | **PCR Efficiency** |
| --- | --- | --- | --- | --- | --- | --- |
| Ribosomal protein L8 | *rpl8* | GGTGCGTCCCTCCTGATG | GCGTGGTGTGGCTATGAAC | 93 | 60.5 | 2.059 |
| Beta tubulin | *tubb4* | TCTTCAGACCAGACAACTT | CTCCTTCCTCACCACATC | 119 | 60.0 | 2.025 |
| Ubiquitin | *ubc* | GGAGGGCAGTAAAGTGATT | CAAGGCAGGAGATTCAGTT | 161 | 57.0 | 1.967 |
| Metallothionein 1 | *mt1* | CCCCTGCTGCCCGACTG | TGTTCAAACTGCCGCCATCTC | 137 | 63.0 | 2.119 |
| Catalase | *cat* | CCAGAAGCGTAATCCTCAA | GAACAAGAAAGACACCTGATG | 100 | 59.0 | 2.071 |
| Hypoxia inducible factor 1 alpha | *hif1α* | GGCAATGGAAGACTTGGA | TGGACTGGAGAACCTTGA | 135 | 60.5 | 2.083 |

**Table S2**: Measured concentrations of copper in the exposure water. Concentrations were measured for three replicate treatment tanks on days 1 and 3 using ICP-MS and are presented as mean values ± SEM.

| **AS Treatment** | **100%** | | **75%** | | **50%** | |
| --- | --- | --- | --- | --- | --- | --- |
| **Nominal Concentration** | **0 μg Cu/L** | **20μg Cu/L** | **0 μg Cu/L** | **20μg Cu/L** | **0 μg Cu/L** | **20μg Cu/L** |
| Day 1 | 0.432 ± 0.024 | 21.634 ± 0.206 | 0.4801 ± 0.022 | 22.943 ± 0.371 | 0.430 ± 0.041 | 21.302 ± 1.163 |
| Day 3 | 0.544 ± 0.026 | 21.837 ± 0.247 | 0.493 ± 0.034 | 22.359 ± 0.763 | 0.446 ± 0.030 | 22.533 ± 1.532 |
| **Mean** | **0.488 ± 0.027** | **21.735 ± 0.151** | **0.487 ± 0.019** | **22.651 ± 0.401** | **0.440 ± 0.023** | **22.071 ± 1.017** |
